# Supplementary material for: Prevalence of dermal trypanosomes in suspected and confirmed cases of gambiense human African trypanosomiasis in Guinea
Source: PLoS Negl Trop Dis. 2024 Aug 19;18(8):e0012436. doi: 10.1371/journal.pntd.0012436 (PMC11361743; doi:10.1371/journal.pntd.0012436)
Supplement: Attachment — Submitted filename: Response letter V1.docx [file pntd.0012436.s003.docx]

**Point-by-point response letter**

We are happy to read that all reviewers appreciated our manuscript. We thank them for their careful reading and their precise and useful suggestions. We have addressed their comments in the point-by-point response letter hereafter. The corresponding modifications are highlighted in yellow in the track-changes manuscript.

EDITORIAL EVALUATION

Address Novelty: Clearly articulate how this study advances beyond the authors' previous work in Clinical Infectious Diseases. Highlight any new findings, methodologies, or insights that distinguish this study.

The study design and most of the methods used in this study are indeed similar (but not identical) to those employed in a previous study (reference 4, Camara et al., Clinical Infectious Diseases 2021). The main differences are (1) the use of a different skin sampling procedure, (skin snip instead of punch biopsy), (2) the inclusion of another distinct active transmission focus (Boffa in addition to Forecariah), (3) the use of a robust and standardized semi-automatized IHC protocol for slide staining and image acquisition and analysis (Zeiss AxioScan + Zen), (4) the longer duration of the follow-up (up to 3 visits over 4 years, instead of 2 visits), (5) the higher number of participants (35 new confirmed HAT cases instead of 18 previously). Fieldwork is tedious and, for obvious ethical reasons, our margin of action was limited regarding the access and use of precious skin samples. Hence, following a protocol comparable to that of our previous study was decided on purpose in order to increase our knowledge and refine our observations in a stepwise manner by fairly comparing novel and previous data. Nevertheless, this new study unveils important findings concerning the detection of trypanosomes in the skin of HAT cases, offering insights that could significantly influence disease management and strategies for elimination.

Clarity and Interpretation: Clarify definitions (e.g., seropositive non-confirmed individuals), improve labeling in figures, and provide clearer interpretations of results, as suggested by Reviewer 2.

We have improved these different points.

Comparative Analysis: Address why certain clinical parameters are not statistically significant and compare with previous studies, as requested by Reviewer 2.

We have addressed these different points.

Data Availability: Consider making individual patient data available for transparency, as suggested by Reviewer 2.

The complete and anonymized raw data base resulting from the entire study is now available in Table S1.

Conclusion: While the manuscript presents valuable findings on the presence of trypanosomes in the skin and its implications for HAT control, it is crucial to address concerns regarding novelty and overlap with previous publications. The editors encourage the authors to revise the manuscript to clearly differentiate it from their previous work and to enhance clarity and interpretation of the findings.

We have improved these different points.

METHODS

Reviewer #3: The methods employed in this study are virtually identical to those employed by the same authors in their previous study (reference 4, Clinical Infectious Diseases 2021;73(1):12–20 ). The only difference in this study was the use of a different skin sampling procedure, i.e. a skin snip versus a blood-free skin punch biopsy. This study also included another active gHAT focus, Boffa, located about 100 km north west of the previously investigated focus of the Forecariah district of the republic of Gambia. As in this previous study the methods and their description are sound.

The study design and most of the methods used in this study are indeed similar (but not identical) to those employed in a previous study (reference 4). The main differences are (1) the use of a different skin sampling procedure, (skin snip instead of punch biopsy), (2) the inclusion of another distinct active transmission focus (Boffa in addition to Forecariah), (3) the use of a robust and standardized semi-automatized IHC protocol for slide staining and image acquisition and analysis (Zeiss Axioscan + Zen), (4) the longer duration of the follow-up (up to 3 visits over 4 years, instead of 2 visits), (5) the higher number of participants (35 new confirmed HAT cases instead of 18 previously). Fieldwork is tedious and, for obvious ethical reasons, our margin of action was limited regarding the access and use of precious skin samples. Hence, following a protocol comparable to that of our previous study was decided on purpose in order to increase our knowledge and refine our observations in a stepwise manner by fairly comparing novel and previous data. Nevertheless, this new study unveils important findings concerning the detection of trypanosomes in the skin of HAT cases, offering insights that could significantly influence disease management and strategies for elimination.

CONCLUSIONS

Reviewer #3: The main result is that immunohistological/PCR methods revealed a significant positive testing for infection when applied to skin samples from seropositive but unconfirmed individuals that was not detected when applied to the blood samples. Interestingly, this positivity in skin biopsies correlated well the clinical manifestations of infection (Table 5). So seropositive individuals who did not show infection by typical parasitology tests on blood, were infected and exhibited clinical signs of infection but the problem of a clinical parameter based diagnosis is the comparable high level of positivity in the seronegative population when compared to the PCR/IH on blood or skin of the group (essentially zero). The authors make the point that there is a need to develop less invasive field adapted methods to detect extravasulature trypanosomes given the fact that these compartments are relevant for infection and transmission. On this point the authors may not be aware of a recent publication, (Larcombe SD et al., 2023, PNAS v120, e2306848120) which shows that proliferating parasites are apparently scarce in the blood after infections are established and that this population might overwhelmingly adapted for transmission. It might be worth considering the relevance of issue of slender/stumpy forms in terms of dermal v bloodstream populations. It is hard to say but the images in Fig 2 look like slender forms. Why not try PCR on a stumpy marker such as PAD1, this might add another element to the results to distinguish them from their previous publications.

We thank the reviewer for these accurate comments. The development of several less invasive extravascular dermal trypanosome detection approaches for HAT diagnosis, as well as the evaluation of the SL/ST proportions among extravascular dermal trypanosomes in human infections are currently being investigated by our teams and will be the object of specific articles in the coming months.

EDITORIAL AND DATA PRESENTATION MODIFICATIONS

Reviewer #1: I have a few minor points that I spotted when reviewing the manuscript.

Line 53-54 – It was not clear how the lower sensitivity of skin snips was critical for detecting trypanosomes in the basal dermis – can you rephrase?

Line 140 – should this read skin snip biopsy not skin snip punch biopsy?

Line 241 – delete ‘of prospections’

Line 241 – should be ‘participate in’

Line 245 – should be ‘to a lesser extent’

Line 260 – should be ‘3 subjects positive’

All changes were done.

I couldn’t find a figure legend associated with the image of the hospital and the additional images of the trypanosomes found by histology.

There was indeed a mistake in the .tif version uploaded on which some unnecessary extra images were appearing. This was fixed.

Reviewer #2: This paper describes a significant prospective observational cohort study aimed at assessing the presence of parasites in the dermis of humans living in areas where HAT transmission remains detectable. This is an important question because, to achieve HAT elimination as established by the WHO, it is vital to identify and monitor all parasite reservoirs. A previous study by these authors demonstrated that T. b. gambiense could be detected in the skin of patients (10.1093/cid/ciaa897). Here, the authors provide a larger-scale prospective observational study (initial screening of 18,916 individuals) in two regions of Guinea. The authors collected clinical, epidemiological, and parasitology data, allowing the classification of individuals as seronegative, seropositive, stage 1, or stage 2. Multiple tests were used to detect parasites, including PCR, immunohistochemistry, CAT assay, and trypanolysis. The authors observed that among confirmed cases, 71% showed parasites in the skin, and among seropositive non-confirmed cases, this number was still quite high (41%). These results suggest that the presence of parasites in the dermis is common, and even when parasites are not detected in the blood, they may be present in the skin. Similar observations have been recently described for extravascular trypanosomes in animals (DOI: 10.1186/s13071-024-06277-7). Importantly, 6-12 months after the initial screening and treatment of all confirmed cases, 25% of seropositive non-confirmed cases still showed parasites in the skin. These findings suggest that, diagnostic methods should be improved to detect skin parasites, and perhaps treatment of seropositive individuals should be considered. I have no major concerns regarding the research described here.

Below are minor points to improve clarity:

- Could the authors label the histology images to indicate the epidermis and the dermis?

A legend was added.

- For a non-specialist in the diagnosis and treatment of HAT cases, it is not clear what a seropositive individual is (someone infected in the last X months; is X known?). It should also be clearer in the Results section that a seropositive individual without detectable parasites in the blood and CSF is considered “seropositive non-confirmed” and is not treated. This is important for understanding the implications of the various numbers described throughout the text.

These important definitions were more clearly explained in the dedicated section of the methods.

- I recommend that in the Results section, description of data is followed by suggestions of its meaning. For example, the paragraph 232-236 could end with a sentence stating, “These results suggest that the presence of one dermatological sign is highly correlated with an active HAT infection or seropositive non-confirmed.”

Some sentences highlighting the clinical result meanings were added when appropriate.

- In the Discussion, could the authors comment on why most clinical parameters are not statistically significant and how this compares with previous studies (doi:10.1371/journal.pntd.0002003)?

The proposed reference is now cited. In this reference, most clinical signs/symptoms were scored from subject reports that could be subjective and then grouped by system/function. The groups of clinical items that were significantly associated with confirmed cases were the occurrence of itchy skin, neurological problems and weigh loss. In the present study, pruritus (itchy skin), circadian rhythm disruptions (neurological problems) and, to a lesser extent, weight loss, were also similarly associated with the disease. Note that the occurrence of swollen lymph nodes is a typical sign observed mostly in Western and Central Africa. Most clinical parameters were not statistically significant simply because, as in previous studies, they were not strongly associated with the disease that can produce a panel of different non-specific signs and symptoms according to the epidemio-immuno-genetic status of the patient.

- Will data form individual patients be available for download? Table S1-S3 has only one tab.

Thank you for this important work and for considering the points above.

The complete and anonymized raw data base resulting from the entire study is now available in Table S1.

Reviewer #3: Specific comments on text

Table 2, a legend would be helpful. The follow up 1 & 2 details are described in the results but should be included here too. In the results the authors mention a decreasing number of subjects for follow up and give reasons for it. However, the figures in the Table are potentially confusing. For example the total number of seropositive unconfirmed (32) and confirmed stage 1 (10) and stage 2 925) add up 67, which is the same number of as the combined totals for follow up 1 (55) and 2 (12). This might be confusing. Maybe be clear that the 55 in follow up 1 is out of the total of 67 that were enrolled as either seropositive and confirmed, and that this dropped to only 12 ex 67 in follow up 2.

For more clarity, an explanation is now provided in the legend of this table.

Table 3. It should be acknowledged that the main clinical parameters, swollen LN, Pruritis, Dermatitis) give almost as good a rate of indication of infection in seropositive unconfirmed individuals as the TBR-PCR and IH on skin samples (table 4) but of course this is qualified by the high rate of similar positivity in the seronegative group. (maybe this is in the text but if so it was no so exlicid).

A sentence was added in the result section to highlight this observation: ‘These results suggest that the presence of one dermatological sign is highly correlated with an active HAT infection.’

Table 5 and P11.

The following section was confusing.

Regarding stage-2 patients followed-up at 6-12 months after treatment (Table 5): among the 3 subjects positives in PCR on skin, two were also found positive in trypanolysis and with a CATTp positive at 1/2 dilution, and the third one was positive in trypanolysis, IHC and with a CATTp positive at 1/8 dilution. The second stage-2 patient positive in IHC was also found positive in trypanolysis and with a CATTp positive at 1/16 dilution.

Should this read The “third” stage-2 patient?

This was clarified.

More generally are the results for these specific individuals, and similarly for those in stage 1, which are described in the text also contained in the figures for the trypanolysis assay for example for stage 8 ex 15 for all VATs. This suggests that individuals who are not positive by TBR PCR are actually positive for circulating antibodies against the VATs.

No, there was no obvious correlation between these two parameters. Out of the 7 stage 2 individuals who were NOT positive by TBR PCR at baseline, 5 were found positive for circulating antibodies against all VATs. In general, serological parameters evolve more slowly and last for longer than parasitological and molecular parameters.

It is not clear either why the number of individuals screen varies in these cases. For example in the trypanolysis assays for Stage 2 at follow up 1 there were 15 screen but 22 individuals of this group were screen ed in the TBR PCR skin assay. It is also odd that the numbers given in the enrolment in Table 5, e.g for stage 1 it is 8 when looking at clinical parameter, differs for that given in table 3, where a number of 10 is given for this group. The same is true for other groups of the seropostives ..for example 24 in T5 v 32 in T3. Why is there a difference between these figures, its not clear from the text.

It is true that, for logistical reasons (human resource variability, limited access for monitoring and limited access to some material and reagents during the 4 years encompassing the COVID pandemics), some few samples were only partially or not collected, and some few analyses were not performed on all samples. This is exactly why we clearly mention the actual denominator for each test.

Minor points, it would be helpful to explain briefly what ISG65 is and why it was included.

This is stated in Material section: ‘Sections were immunolabelled with the *T. brucei*-specific anti-ISG65 antibody which targets the Invariant Surface Glycoprotein 65 (rabbit 1/800; gift from M. Carrington, Cambridge, UK) [15].’

VAT, maybe just a note to say same as VSG?

This is now better explained in the ‘Immune trypanolysis test’ section.

SUMMARY AND GENERAL COMMENTS

Reviewer #3: It seems to this reviewer that this study is more or less repeats the work described in a previous study same authors (Ref 4). The results are also similar and support the view that the skin is a significant reservoir of extravascular parasites and that trypanosomes are present in this compartment in undiagnosed/unconfirmed individuals. They also present bring evidence from foci of infections in the field that support, align with ideas of the initiating study that highlighted which identified the skin as a significant reservoir of trypanosomes that goes undetected by typical bloodstream parasite assessments (Ref 2). In turn these skin resident trypanosomes, which are not detectable by the typical bloodstream based diagnostic approaches could contribute to disease transmission. Overall, the results and the data support the arguments and views of the authors in their discussion. It is also nice to see publications reflecting HAT in the field, so there is a direct relevance to the study for transmission and disease elimination strategies. Having said that, there is no doubt that the results are very similar in content, outcome, and impact to that of a previously published study by the same authors (Ref 4).

See our response above.
